# Corneal elastic property investigated by terahertz technology

**DOI:** 10.1038/s41598-022-22033-2

**Published:** 2022-11-10

**Authors:** Lin Ke, Lei Zhang, Nan Zhang, Qing Yang Steve Wu, Hai Sheng Leong, Ali Abdelaziem, Jodhbir S. Mehta, Yu-Chi Liu

**Affiliations:** 1 Institute of Materials Research and Engineering, Agency for Science, Technology and Research, 2 Fusionopolis Way, #08-03 Innovis, Singapore, 138634 Singapore; 2 National Institute of Laser Enhanced Sciences (NILES), Cairo University, Giza, 12613 Egypt; 3 Singapore Eye Research Institute, Singapore, Singapore; 4 Singapore National Eye Centre, 11 Third Hospital Avenue, Singapore, 168751 Singapore; 5 Duke-NUS Medical School, Ophthalmology and Visual Science Academic Clinical Research Program, Singapore, Singapore

**Keywords:** Biophysics, Medical research, Materials science, Optics and photonics

## Abstract

Terahertz (THz) spectroscopy technique has been applied in ex vivo biomechanical properties analysis of human corneas. Upon the application of light pressure on the cornea, the photo elastic birefringent effect, anisotropic deformation, thickness changes and hydration levels will contribute to the sudden phase changes of terahertz time domain signal. The shelf lifetime study shows that the phase shift is reduced and cornea loose the biomechanical properties with the increase of hydration level. Mechanical behaviors have been further studied based on the “fresh” cut corneas with the similar hydration levels. THz signal was collected by focusing inside of the cornea to avoid the phase shift due to light stress caused movement of the corneal surface. By this way, the amount of THz signal refractive index variation is correlated to the elastic property of the corneas. The correlation between the THz signal phase shift and refractive index shift due to the corneal strain can be used to derive the elastic Young’s modulus. Our results demonstrated the THz spectroscopy, as a non-contact and non-invasive detection method, could be potential for understanding the mechanism of corneal deformation under the action of intraocular pressure in the physiological environment in future.

## Introduction

As a viscoelastic tissue, cornea exhibits complicated biomechanical properties, such as: nonlinear elasticity, anisotropy, and viscoelasticity. The biomechanical properties play an important role in keeping the normal structure and function^1^. The immediate mechanical environment of underneath the epithelium directly affects the cellular stiffness of limbal epithelial stem cells (LESCs) which responsible for proliferation and differentiation to repopulate the central corneal epithelium^2^. Furthermore, refractive and therapeutic treatments, ocular or systemic diseases may induce changes in the mechanical resistance of the cornea^3^. Biomechanical property changes due to surgical incision and laser ablation introduce certain degree of corneal expansion affecting correction results after refractive surgery^4–6^. Changes in biomechanical properties are always earlier than the clinical symptoms, therefore, increasing interests have arisen in the measurement of corneal biomechanics to predict corneal response to surgical or therapeutic interventions^7^. However, it is difficult for ophthalmologists to understand the mechanism of corneal deformation under the action of intraocular pressure in the physiological environment due to the lack of in-vivo corneal biomechanical performance parameter detection technology^8^.

Intraoperative anterior chamber manometry^9^ is an existing cornea rigidity test method based on the enucleated eyes. It may not reflect the intraocular pressure in the real situation. Axial length (AL)-associated Ocular Rigidity (OR) measurement^10^ is based on the ocular AL changes, induced by the oral administration of acetazolamide, measured with coherence laser interferometry. This measurement process takes long time and causes the patients suffering. Ultrasound elastography^11^, which is reported to be potential used as a non-destructive measurement tool for real-time in vivo measurements of OR. Currently there is no commercial products for in vivo measurement yet. Corneal hysteresis, measured with the Ocular Response Analyzer (ORA, Reichert Inc., Buffalo, NY, USA), which reflects viscous damping in the cornea. The capacity of corneal tissue to recover its shape following the application of external forces^12,13^. This measurement process is intrusive, time consuming and causing inconvenience for patients and clinicians. Optical coherence elastography (OCE), which is another emerging non-invasive elastography technology based on OCT and can quantitatively measure the elastic properties of the cornea^14^. OCE obtains the elastic properties of the cornea by measuring the longitudinal vibration of the cornea, the propagation velocity of shear waves or the propagation velocity of surface waves caused by external or internal excitations^15–17^. The relationship between corneal classical mechanical property e.g. Young’s modulus, force, displacement and the dynamic OCE techniques have not yet been set up which hindered the technology adoption. Brillouin spectro-microscopy (BSM) is an instrument integrates phased array spectrometer with optics interferometric filter, which has the capabilities of measuring the relative stiffness of different areas of human corneas, thus providing a non-contact method to characterise the fundamental mechanical features and has consistently demonstrated that corneal biomechanics is highly heterogeneous, and shows considerable variations between its centre and periphery as well as between its anterior and posterior regions^18,19^. However, BSM has weak signal, which entails relatively long data acquisition times and potentially harmful illumination dosages. This has often limited Brillouin microscopy to ex-vivo samples or relatively static biological conditions. The abovementioned methods are either intrusive, time-consuming, causing pain to the patients or premature technologies which are not ready for in vivo measurement.

Terahertz (THz) technology has been shown as a safe evaluation tool for ocular tissue^20–23^. It is non-invasive, non-ionized, label-free and sensitive to the hydration and collagen composition changes^24^ which demonstrated vast potential applications in biological area^25^. THz also demonstrated the capability of biomechanical analysis for various other tissues and biomaterial structures^26,27^. The cornea is suitable for THz scanning as the cornea consists primarily of water (78%) and scientists all over the world attempted to use THz technology in ophthalmology applications^28–31^. However there is no reports of using THz technology for corneal mechanical parameter extraction. In this paper, visible range wavelength “light” was used to trigger the elastic changes of the cornea and THz beam was used to observe the structure changes. The method is proved to be effective and useful and there is no prior report in this field so far to our knowledge.

## Experimental setup

### Sample preparation and thickness measurement

Three batches, each batches contains two human cadaveric corneas, have been procured from overseas eye banks (Lions Eye Bank, Rochester, NY, USA) after donor consents were obtained. The use of cadaveric donor corneas for this study was reviewed by Singhealth Centralized Institutional Review Board, and exempted from the need of approval as no identifiable data were used in this study. All imaging evaluation performed involving these donor corneas were carried out in accordance with the tenets and the principles outlined in the Declaration of Helsinki. For this study, all research grade cadaveric donor corneas were procured from Lions Eye Bank (Rochester, NY, USA) with informed consent from the next of kin.

These corneas had different death-to-preservation time and different preservation time before arrival, hence represented with different elastic properties.

The corneas were scanned by the RTVue ASOCT (Optovue, Inc, Fremont, USA). Three high-resolution corneal cross-sectional scans (8 mm scan length, single scan mode) were obtained for each sample at each time instant^32^. The central corneal thickness was measured using the built-in software callipers at the corneal center as we published^33^.

Currently, the reported power for human eye imaging using OCT is around 200 µW^34^. To ensure the safety of using light stress, the following experimental conditions have been designed and executed. One batch of two corneas undergone light stress of 633 nm with power of 55 µW, one batch of two corneas undergone light with wavelength of 532 nm with different light stress power of 55 µW and 45 µW; one batch of two corneas under 633 nm and 532 nm with power of 45 µW.

The laser power has been measured using Melles Griot power meter, 13PEM001, which is calibrated within the spectral range of 400 nm-2 µm. The experiments have been repeated with different stress time for each samples of 10 s, 20 s and 30 s. Table 1 shows the information of the samples. The mechanical behavior of corneas has been studied with different hydration levels. One typical cornea sample was measured using THz spectroscopy at different observation period of 1 day, 4 days, 7 days and 10 days. The thickness of the corneas measured along the observation period can be used to indicate the different hydration levels. Thereafter two corneas from Batch 1 which have the similar initial thickness and the same stress conditions, have been used to study the mechanical performance. We will report the detailed experimental results based on the batch 1 samples and finally an overall conclusion will be given based on all the samples.

**Table 1:** sample thickness and stress parameters of the laser light.

Batch number	Sample number	Initial thickness (µm)	Wavelength of light (nm)	Light power (µW)	Light pressure (10^−8^ Pa)
1	1	581.65	633	55	3.67
	2	578.13	633	55	3.67
2	3	583.25	532	55	3.67
	4	598.75	532	45	3.00
3	5	681.37	633	45	3.00
	6	552.78	532	45	3.00

### Light stress optical system

The beam size has been focussed to 5 mm^2^ in the sample for both 633 nm and 532 nm laser used in this experiment. Based on the laser light wavelength, power and beam size, the pressure *P*_*s*_ exerted on the corneal samples can be calculated as:

1 $$P_{s} \; = \;\frac{I}{C}\; = \;\frac{{{\raise0.7ex\hbox{$P$} \!\mathord{\left/ {\vphantom {P A}}\right.\kern-\nulldelimiterspace} \!\lower0.7ex\hbox{$A$}}}}{C},$$

where I is the laser intensity, P is the laser power, A is the beam size area of light source and *c* is the speed of light in vacuum. Light pressure remains unchanged during the experiment. THz spectral has been collected continuously during the presence of light stress. The light pressure data are indicated in Table 1.

### THz spectroscopy system

**Figure 1:**
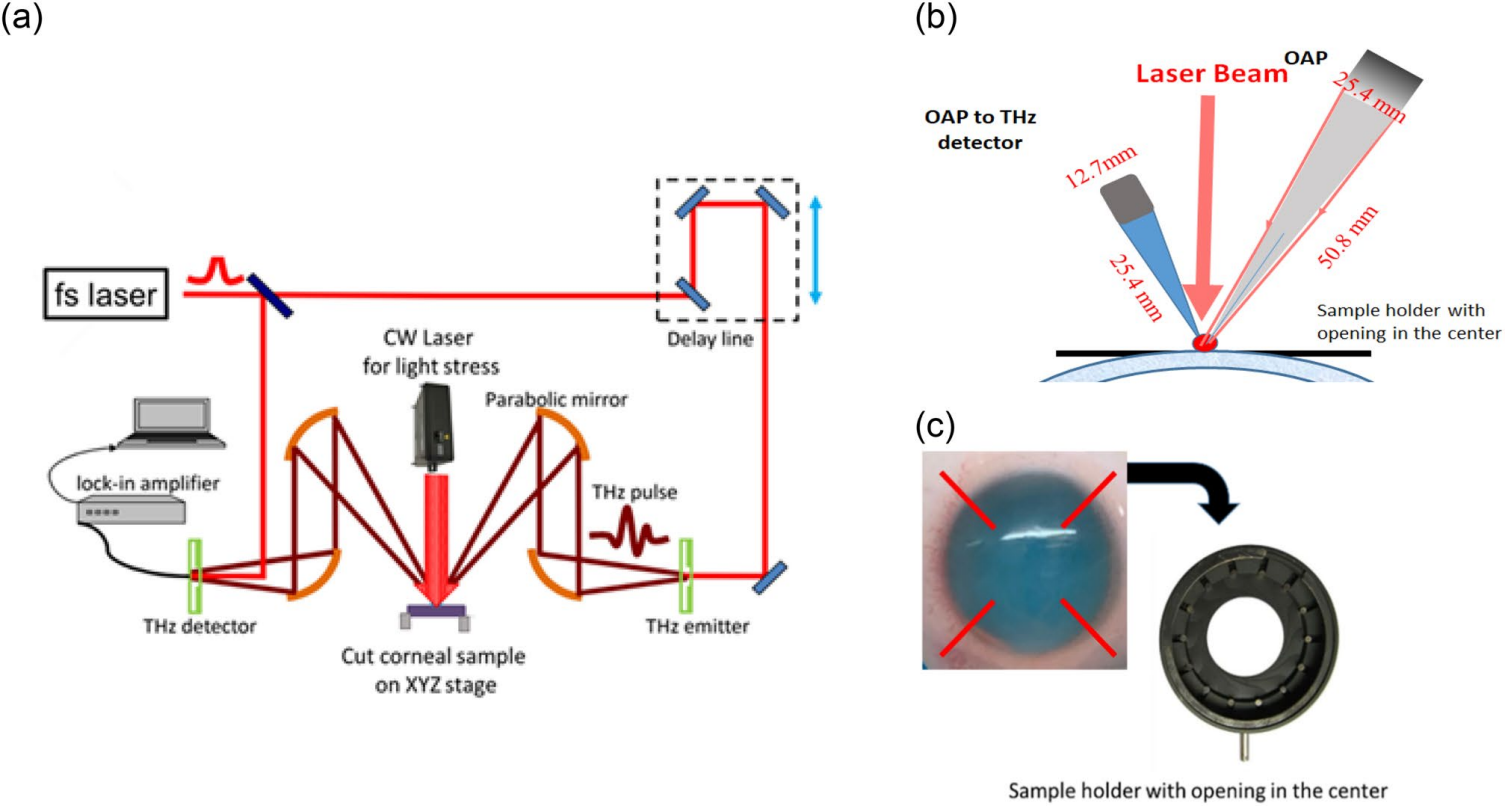
(**a**) Schematic THz spectroscopy measurement setup and light stress executed on top of the corneal sample. (**b**) Detailed information of parabolic focusing setup. (**c**) Sample has been flattened on holder with opening on the XYZ stage.

TERA K15 (Menlo Systems GmbH, Germany) was used in this experiment as depicted in Fig. 1 (a). In the system, two femtosecond fibre lasers with 250 MHz repetition rate and 90 femtosecond laser pulses with around 1.56 μm central wavelength are used to excite two photoconductive antennas namely emitter and receiver with the coverage of bandwidth up to 3.5 THz. THz source beam is collimated by a 76.2 mm effective focal length (EFL), 25.4 mm clear aperture off-axis 90° parabolic (OAP) mirror. A 50.8 mm EFL OAP mirror at a 30° incidence angle is used to focus the beam onto the cornea surface as shown in Fig. 1b. The incident THz wave is linearly polarized with a tilted angle 45 degrees to the vertical direction, the output THz wave reflected from the sample is passed through an analyzer with a transmission direction tilted at an angle of 90 degrees to the polarizer. TERA K15 system stability has been studied for more than 4 h using single shot mode in ambient air. The overall THz spectrometer pulse-to-pulse peak jitter is < 5 fs.

The THz system was calibrated by maximizing reflectivity off a piece of aluminium sample surface. The samples were mounted on the XYZ stage. At first, the beam from the emitter focuses on the cornea far front surface through parabolic mirrors as indicated in the Fig. 1b. Focusing has been fine-tuned to achieve the highest time domain spectra (TDS) intensity, the 1st reflected THz signal has been collected. The laser stress was applied at the same detection point, the 2nd reflection THz signal was collected. The location of corneal sample is fixed for both conditions—with external light and without external light on to remove the sample position caused phase shift. The corneal mechanical parameter extraction is greatly affected by the measurement techniques, the refractive index, thickness, and position for the samples are all resulting in the phase shift. By the measurement setup as indicated in the Fig. 1b, we could reduce the sample position caused phase shift. The THz time domain amplitude and phase changes will be considered from photoelastic changes and thickness changes of the corneal samples.

The corneal samples were further cut open from the corner along the red color lines as depicted in Fig. 1c, so that the cornea samples can be flattened on the sample stage. The sample is standalone with air in both front and behind. To ensure the accurate measurement of the phase of the reflected signal (by monitoring the relative timing of the signal reflected from the sample compared to the reflection from the first surface of the window), enhance the reflection signal intensity, and reduce scattering effects. The samples must be flattened to reduce the phase errors due to the position changes. The samples in our experiments are put in the sample holder and the central part is free standing. This is to simulate more realistic situation. The free-standing configuration without additional apparatus or fixtures make it easier for subsequent Young’s modulus calculation.

### Theoretical calculation

For human cornea, the elastic modulus is the main mechanical characterization parameter to indicate the anterior and posterior corneal shapes. Understanding the corneal shape and thickness and their relationship with the tissue's mechanical properties is important to refractive or therapeutic corneal treatments. In the process of elastic deformation of corneas, the proportional relationship between stress and strain is Young's modulus, also known as elastic modulus. According to Hooke's law, within the elastic limit of an object, the elastic (Young's) modulus, the stress and strain has the relationship of E = σ/ε. In this work, light pressure is applied to cornea, the dielectric constant (refractive index) changes owing to the photoelastic effect, the mechanical properties of the corneas will be studied.

Utilizing a polarized THz light beam to study the stress-optical effect in THz regime and detect the stress states were reported by many researchers^35,36^. Cornea sample is strong dispersive; phase retrieval has to be done correctly in order to have the correct extraction of the complex-valued dielectric properties^23^. Moreover, the Beer Lambert Model cannot be used for assessments in the case of a heterogeneous environment, which is obviously exists in such a complex structure of the cornea. To extract the cornea property parameters, a numerical fitting technique is used.

This technique first uses a theoretical model (Cole-Cole model or Cole-Cole Drude model) that mimics the multilayer of the corneal samples: the epithelium, stromal and endothelium to simulate the reflected spectrum. An optimization algorithm compares the simulated reflected spectrum to the empirical one and then proceeds to minimize the difference between them by selectively altering the parameters in the theoretical model. Once the error is minimized, the refractive index from this set of optimal parameters are concluded as the actual refractive index of the cornea.

The stress-optical law states that the mechanical stress makes an originally isotropic material become optically anisotropic and the optical axes of the stress induced birefringence are aligned with the principal stresses’ axes. Figure 1 shows schematic THz spectroscopy measurement setup and light stress executed on top of the corneal sample.

If the specimen is under the plane stress state, the refractive index variations ΔN can be expressed using the stress parameters according to the following equation^36,37^:

2 $$\Delta \mathrm{N}=\frac{c\left({\delta }_{1}-{\delta }_{1}^{^{\prime}}\right)}{2\pi fd}=\frac{c\Delta \delta s}{2\pi fd},$$

where ΔN represents the refractive index changes measured by the THz spectra, $\Delta {\delta }_{s}$ is the phase delay changes caused by stress, f is the frequency of the THz radiation, c is the speed of light in the vacuum and d is the original thickness of the specimen.

The phase delay of the terahertz pulse is mainly caused by two factors, one is the change in refractive index caused by the law of photoelastic effect and the other is the change in thickness caused by the Poisson’s effect.

The measured phase delay in the experiment has two parts, $\Delta {\delta }_{s}$ and $\Delta {\delta }_{d}$, therefore, we further extend the phase delay to include both thicknesses induced delay and stress induced delay:

3 $$\Delta\updelta =\Delta {\delta }_{s}+\Delta {\delta }_{d}$$

It is known that the thickness of the specimen changes under stress, which can be expressed as

4 $$\Delta \mathrm{d}=\frac{d\cdot \mu \cdot \sigma }{E},$$

where μ, σ and E are Poisson’s ratio, the interior tensile stress, and elastic modulus, respectively. The thickness change can be observed from the time domain spectral. Supposing the initial refractive index of the corneal is N_0_, the phase change of $\Delta {\delta }_{d}$ induced by the decrease of thickness can be written as follows^35^:

5 $${\Delta \delta }_{d}=\frac{2\pi f\left({N}_{0}-1\right)\Delta d}{c}$$

Therefore, the phase change induced by the stress can be further corrected as:

6 $$\Delta \mathrm{N}=\frac{c}{2\pi d}\frac{\Delta \delta }{f}+\frac{\left({N}_{0}-1\right)\mu \sigma }{E}$$

From THz time domain spectroscopy, we can obtain the phase delay and refractive index changes, Young’s modulus can be extracted.

The corneal hydration change will affect the corneal mechanical property by introducing extra phase changes in the THz time domain spectra. In the first part of the work, the corneas THz time domain spectra have been collected and analysed for 10 days to observe the hydration level changes contribute to the significant time-domain phase changes. However, in the 2nd part of the work, we will focus on the extraction of the mechanical parameters of the cornea at the same corneal hydration level. The theory and experimental work of corneal hydration effects on the mechanical parameters’ calculation will be subjected to further study.

## Results and discussion

### Stress and hydration effects on the THz time domain spectral behaviour

**Figure 2:**
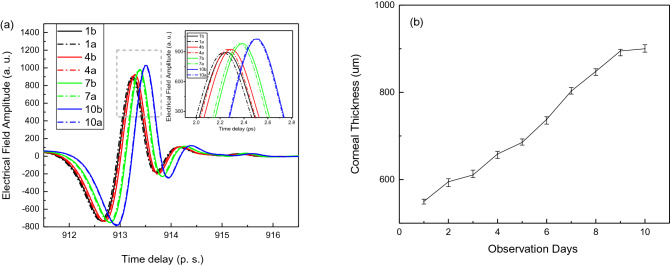
(**a**) Time domain spectra obtained at day 1, day 4, day 7 and day 10. Inset figure: enlarged peak information (**b**): thickness data with error bar indication versus observation days.

The hydration effects on the cornea mechanical performance have been studied. Figure 2a shows one of the typical cornea’s time domain spectra with stress and without stress curves obtained at day 1, day 4, day 7 and day 10. The measurement done after finding the far front surface of the cornea. The 1a, 4a, 7a and 10a indicate the curves after the light stress, while the 1b, 4b, 7b and 10b indicate the curves before the light stress. When the light stress is on, the curves shifted towards to the shorter delay time direction. The reflection intensity slightly decreased. The thickness of the corneal samples has been measured along the observation days and shown in Fig. 2b, the measurement has been repeated several times and the error bars are indicated in the graphs. Figure 2a also demonstrated that with observation time increase, the hydration level will increase, the smaller changes of THz signal observed which reflects the cornea elastic property decreased.

**Figure 3:**
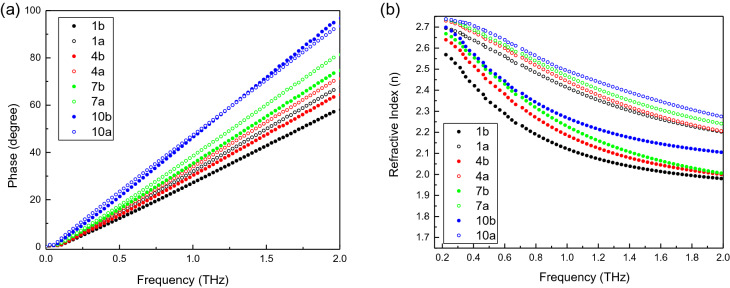
(**a**) Phase versus frequency; (**b**) Refractive index value versus frequency for cornea sample No. 1 at day 1, day 4, day 7 and day 10.

Figure 3a shows the phase versus frequency results for cornea with stress and without stress obtained at day 1, day 4, day 7 and day 10. With the observation time increase, both the cornea thickness and hydration level increased. The phase difference due to light stress decreased. Our results demonstrated that the hydration level changes contribute to the changes of cornea optical and mechanical properties, which lead to significant time-domain phase changes. Figure 3b shows the refractive index versus frequency results for cornea with stress and without stress obtained at day 1, day 4, day 7 and day 10. The hydration level changes contribute to the significant time-domain phase changes and further affect the changes of the refractive index of the corneas which indicates the corneal mechanical properties has been changed. From Fig. 3b, we found that with the observation time increase, the refractive index difference due to light stress decreased. Our results demonstrated that the hydration level changes contribute to the change of cornea optical and mechanical properties.

#### Biomechanical properties of the corneas

Based on the theory derivation, we further analyzed the corneal biomechanical properties by extraction out of the corneal mechanical parameters. We observed that at the initial state of the cornea delivery, the thickness of the corneas sample No. 1 and No. 2 have roughly the similar thickness, though the biomechanical properties test were conducted around the 3rd and 4th day after the cornea delivery due to logistic issue. The thickness values were obtained in SERI before shipping to our institute for THz experiments, the delay of the two experiments around 24 h.

**Figure 4:**
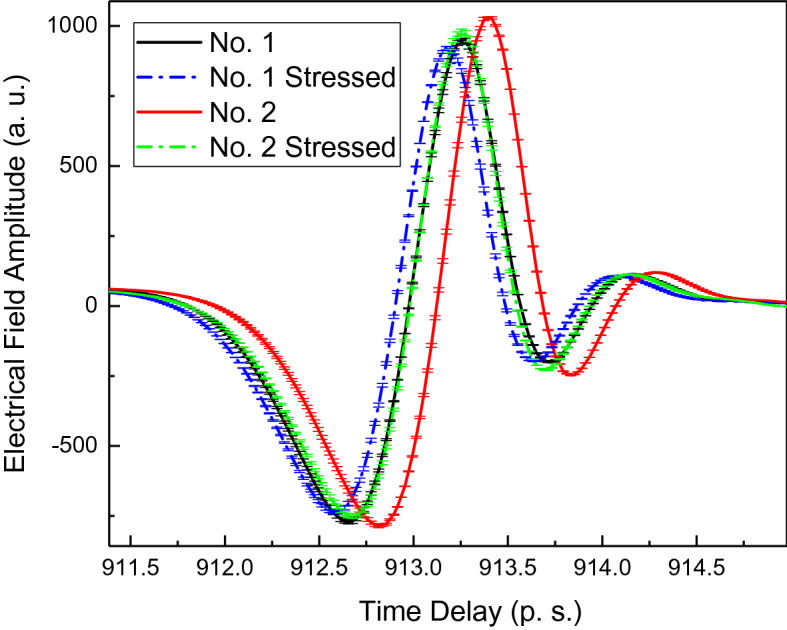
THz time domain spectra collected on the surface of corneas with the indication of data error bar.

Figure 4 shows THz time domain spectra of the two cornea samples before stress and under stress. The THz reflection signal was collected on the corneal surface when no light stress was applied. The THz reflection signal was collected again when the light switched on. The black solid line shows the corneal No. 1 THz time domain spectra (TDS) without light stress. The blue dash line shows the corneal No. 1 THz TDS under light stress; similarly, the red solid line shows the corneal No. 2 THz TDS without light stress. The green dash line shows the corneal No. 2 TDS under light stress. Upon the light was switched on, both corneas’ THz TDS spectra shifted suddenly, both their peak delays and the reflection intensities were reduced as indicated in the Fig. 4.

**Figure 5:**
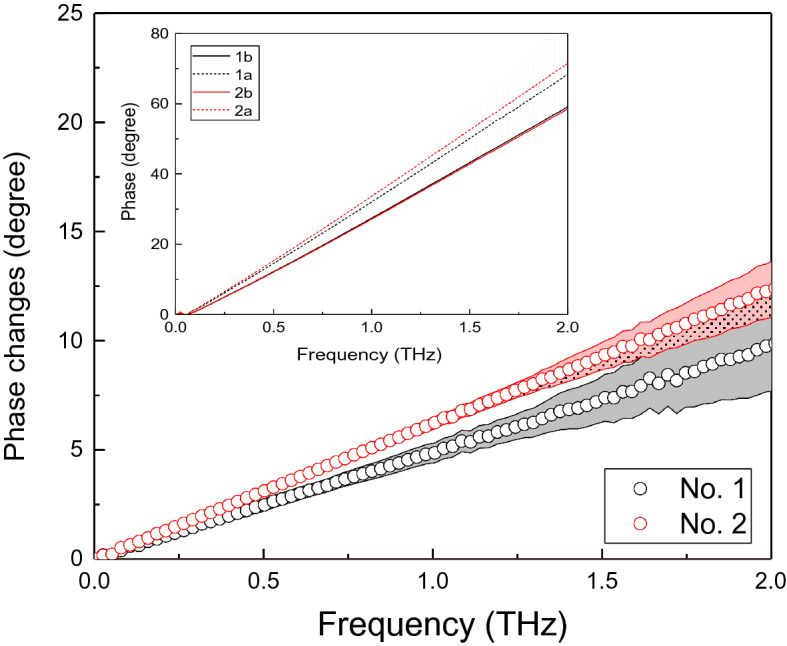
The phase difference versus frequency for corneal samples No 1. and No. 2, data with error bar indicated. Insert figure: Phase versus frequency for cornea No. 1 and No.2. 1a and 2a indicate the curves after the light stress, while the 1b and 2bindicate the curves before the light stress.

Phase versus frequency information has been extracted and plotted in Fig. 5 by Fourier transformation of the time domain spectra presented in Fig. 4.

The phase difference versus frequency for corneal samples No. 1 and No. 2 are shown in Fig. 5. Black curves are for the cornea No.1 and red curves are for the cornea No. 2. Solid lines indicate the data collected when the corneas are not under light stress, while dash lines indicate the data collected when the corneas are under stress.

Across over the observed wide frequency range from 0.1 THz to 2 THz, the cornea No. 1 shows the smaller phase change compared with cornea No. 2. Assuming sample No. 1 and No. 2 have the similar hydration level. The phase difference is from the birefringent effects and the instant thickness change. The mechanical properties, e.g. Young’s Modulus can be extracted. Error bars has been added into the Fig. 5. The errors are increasing with frequency increase.

In order to extract the cornea property parameters, a numerical fitting technique is used to extract the refractive index. This technique firstly uses a theoretical model that mimics the corneal multilayers and hydrogel layer behind to simulate the reflected spectrum. An optimization algorithm compares the simulated reflected spectrum to the experimental result. The difference between them can be minimized by selectively altering the parameters in the theoretical model. The parameters with the best fit will give the absorption spectra, refractive index of the samples.

**Figure 6:**
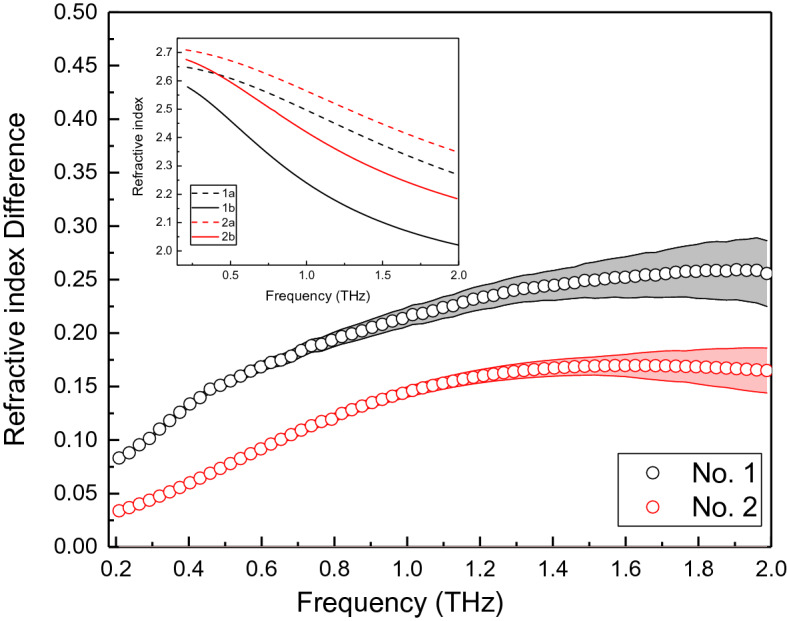
Refractive index versus frequency for the cornea No. 1 and No. 2. Insert Figure: refractive index of cornea No. 1 and No. 2. 1a and 2a indicate the curves after the light stress, while the 1b and 2b indicate the curves before the light stress.

Figure 6 shows refractive index difference versus frequency for the cornea No. 1 and No. 2. Both curves for stress and under light stress are shown. Black curves are for the cornea No.1 and red curves are for the cornea No. 2. Solid lines indicate the data collected when the corneas are not under light stress, while dash lines indicate the data collected when the corneas are under stress. Upon the light stress on, the refractive index for both cornea No. 1 and No. 2 increased. Error bars has been added into the Fig. 6. The errors are increasing with frequency increase.

Cornea No. 1 shows larger shift of the refractive index across the frequency range observed than the corneal No. 2. This indicates that cornea No.1 has greater optical performance changes under the same stress. This is in agreement with the phenomena demonstrated in Fig. 2, the higher changes of THz signal reflect higher elastic property of the corneal samples, the optical parameters also demonstrated higher changes under the same light stress.

**Figure 7:**
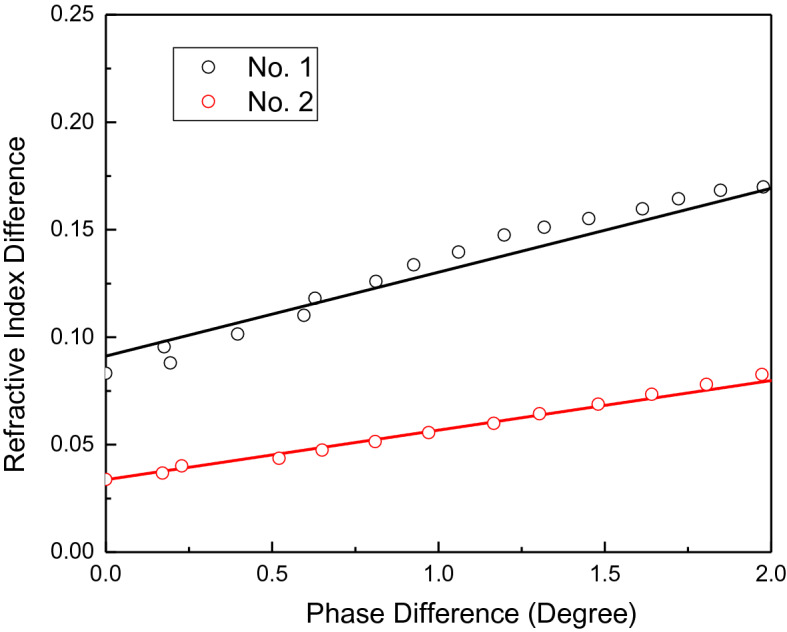
The refractive index difference versus phase shift curves for corneal sample No. 1 and No. 2 at stress duration of 10 s.

From Figs. 5,6, the refractive index difference versus phase shift curves are plotted in Fig. 7. Cornea No.1 has higher values and higher changing slope. This indicates that with the same phase change, the refractive index shift induced is much higher for cornea No.1 than cornea No. 2. The increasing speed is higher for cornea No. 1 too.

Linear fitting is done to calculate the parameters according to Eq. (6). From Fig. 7, the slope of No. 1 and No. 2 are 0.04879 and 0.04243 respectively. The calculations are based on several data collected when the light stress is on. The calculated thicknesses are related to frequency. At frequency of 1.5 THz, the thickness calculated have average values of 636.09 µm and 731.57 µm for corneas No.1 and No. 2 respectively. OCT has been done on the two corneas the previous day before THz measurement at National Eye Center and the thickness are 615 µm and 685 µm. The experiments were carried out around the 3rd and 4th day of the cornea storage day after the delivery. The thickness values we obtained from THz measurement and derivation are comparable with the OCT measurement results.

From Fig. 7, the intercepts of cornea No. 1 and No. 2 are 0.0858 and 0.04544 respectively. The cornea No. 1 has higher intercept value compared with No. 2. The N_0_ is extracted from Fig. 6 inset Figure and frequency at 1.5 THz and Poisson ratio is 0.49^41–43^. Young’s modulus for corneas No. 1 and No. 2 are 0.2207 MPa and 0.4217 MPa, respectively. Cornea No. 1’s Young’s modulus is within reasonable range of cornea’s Young’s modulus as reported in the literature^41–43^, cornea No. 2’s Young’s modulus is slightly higher. The reason could be the No. 2 cornea is not in the good condition or is an aged cornea. Though in the beginning, the fresh cut corneas No. 1 and No. 2 have relative same thickness, the No. 2 cornea thickness increased very fast. The other reason could be the experiments are done in ex vivo environment, which have different physiological environment. There are reports of ex vivo higher value of Young’s modulus^45^ and much higher Young’s modulus value for aged corneas too^44^. Table 2 listed the reported refractive index, Poisson’s ratio, elastic modulus values reported from literature and their resources. Table 3 listed out the parameters used for the calculation and the calculated cornea thickness and Young’s modulus values by the methodology we described.

**Table 2:** The parameters used for calculation of corneal thickness and Young’s modulus.

The refractive index reported	The Poisson’ ratio	Elastic modulus	Reference
2.25 to 3.5 (THz range)			^31,38,39^
1.3–1.4 (visible range)			^40^
	0.49	0.16–0.3 MPa in vivo	^41^
	0.49	0.29 MPa in vivo	^42^
		0.29 MPa in vivo	^43^
		Age 20 years (95% confidence interval, 0.22–0.31) to 0.52 (0.50–0.54) MPa at age 100 years (R^2^ = 0.70)	^44^
		0.656 MPa ex vivo porcean cornea	^45^

**Table 3:** The parameters used for calculation and the derived mechanical parameters related to cornea No. 1 and No. 2.

	Stress duration (sec)	Slope	Thickness	Thickness (average)	Intercept	Young’s modulus (MPa)	Young’s modulus (average)
Eye 1	10	0.04871	637.04	636.09	0.0912	0.2069	0.2207
	20	0.04823	643.38		0.0875	0.2156	
	30	0.04942	627.89		0.0787	0.2395	
Eye 2	10	0.04192	740.22	731.57	0.03943	0.47844	0.4217
	20	0.04213	736.54		0.04367	0.43199	
	30	0.04322	717.96		0.05321	0.35454	

In summary, two cornea samples have been given external light stress and THz spectroscopy have been used to study the optical parameter’s behaviors. Thereafter, the elastic properties of the cornea were studied. Cornea No. 1 shows higher change in time domain spectrum compared with Cornea No. 2 under the same stress condition. The calculated Young’s modulus is smaller for sample No. 1. While cornea No. 2 shows smaller time domain spectrum shift, rigidity bigger and the calculated Young’s modulus is bigger. Two more round of experiments have been done to repeat the experiment with different light stress time. The Young’s modules derived are listed in the Table 3.

This is the first trial of using non-destructive terahertz broadband spectroscopy for study of ex vivo human corneal mechanical properties. The corneal tissue behaviour is much more complicated in real bio-environment in-vivo, however, the biomechanical testing of ex-vivo corneal tissue will contribute the understanding of corneal biomechanics and provide a possible future non-invasive method for in vivo study.

## Conclusion

In this work, THz spectroscopy was applied as a probing method to extract the cornea elastic properties. Light stress was applied on the corneas to generate mechanical deformation, which results in phase change. THz signal was observed to have a sudden change with the application of light pressure. The amount of signal change is correlated to the elastic property of the corneas. According to the deformation of the cornea when the light stress was on, the relationship between the THz signal phase shift and refractive index to the corneal strain is used to derive the Young's modulus. The present approach is novel in the way that it uses light pressure to trigger the deformation changes and uses THz signal to observe the structure change, from which the structure information, e.g. the cornea elastic constant/rigidity parameter information can be calculated. The method demonstrated in this work shows potential for further ophthalmology application. In future, the technique could be applied in practical situation to obtain the in vivo biomechanical properties of corneas in physiological environment.

## Data Availability

The datasets generated and/or analysed during the current study are available from the corresponding author on reasonable request.
